# Alternative Prosthodontic Therapies: A Multifaceted Approach

**DOI:** 10.7759/cureus.29363

**Published:** 2022-09-20

**Authors:** Arushi Beri, Sweta G Pisulkar, Akansha V Bansod, Chinmayee Dahihandekar

**Affiliations:** 1 Department of Prosthodontics and Crown & Bridge, Sharad Pawar Dental College and Hospital, Datta Meghe Institute of Medical Sciences, Wardha, IND

**Keywords:** homeopathy, hypnosis, ayurveda, acupressure, acupuncture, prosthodontics, alternative therapies

## Abstract

The increasing influence of alternative therapies reflects shifting demands and attitudes in modern society in general. Concerns about the negative impacts and rising expenses of traditional health care are also fueling the hunt for alternatives. Acupuncture, acupressure, homeopathy, Ayurveda, and hypnosis are examples of therapy modalities. Alternative therapies, on the other hand, are currently recommended as a supplement to traditional treatment. Although their efficacy as a standalone therapy is debatable, when used in conjunction with conventional treatment, they can be a valuable addition to the general dentist’s therapeutic arsenal. When mainstream medicine cannot give a proper treatment or simply offers symptomatic alleviation for chronic diseases, the patient’s desire for alternative therapies rises. This was found to be true for common medical conditions such as back pain and asthma. Surprisingly, patients seek conventional medical practice treatments for dental or oral health issues more frequently in emergencies (i.e., tooth pain or dental decay, temporomandibular joint disorder, periodontal disease, or dry mouth). Cloves and tea tree oil are the two topical or oral herbal or natural products that dental patients utilize most frequently. Furthermore, people worried about getting dental work done may find great benefits from using mind-body techniques.

## Introduction and background

The growing popularity of alternative therapies reflects shifting demands and attitudes in modern society. Concerns about the negative impacts and rising expenses of traditional health care are also fueling the hunt for alternatives. Treatments that are not traditional (mainstream therapy) are referred to as alternative therapies [1]. According to a European Commission report, “alternative therapy is a broad field of healing resources that encompasses all health systems, modalities, and practices, as well as their supporting theories and beliefs, other than those intrinsic to the politically dominant health system of a given society or culture in a given historical period.” When used properly, these serve as an addition to the dentist’s toolkit and are used to strengthen traditional treatment approaches. Complementary and alternative medicine refers to a variety of medical and healthcare practices, programs, and products that are not generally accepted as a part of conventional medical practice (CAM). The National Center for Complementary and Alternative Medicine (NCCAM) was founded by the National Institutes of Health (NIH) to serve as a public resource for CAM use. In the past 14 years, CAM therapies have attracted increased attention in the United States. According to research, CAM spending and usage increased in the United States by roughly 8% [2]. Complementary and alternative medicine is divided into five categories by the NCCAM. Alternative medical systems, which include whole systems and procedures, come first. Two of these customs originated in Western societies, namely, naturopathic medicine and homeopathy. Traditional Chinese medicine (TCM) and Indian Ayurveda are two very old systems that originated in non-Western cultures. The second area is mind-body interactions, which include music, dance, art, and biofeedback therapies, as well as meditation, prayer, and mental healing. The third group consists of physiological therapies, which include dietary supplements, meals, vitamins, and herbs (e.g., shark cartilage to treat cancer). The body-based and manipulative techniques in the fourth category include massage, chiropractic, and osteopathic manipulation. Energy therapy is included in the fifth category. These consist of bioelectromagnetic-based therapies such as pulsed fields, magnetic fields, and alternating or direct current fields, as well as hands-on biofield therapies including Gi Gong, Reiki, and therapeutic touch. Both TCM and Ayurvedic medicine depend largely on the use of plant-based prescriptions for the treatment of particular illnesses. While using isolated or manufactured single compounds, Western medicine mainly abandoned treatments based on plant extracts. Due to some of the severe side effects associated with the use of some western medications, such as the birth deformities linked to thalidomide, there is currently a tendency toward the usage of these ancient methods. However, if administered incorrectly, plant-based medications can be just as harmful as synthetic drugs [2].

## Review

Types of alternative therapies

Acupuncture, the Alexander technique, aromatherapy, Ayurveda, biofeedback, chiropractic medicine, dietary therapy, herbalism, holistic nursing, homeopathy, hypnosis, massage therapy, meditation, naturopathy, nutritional therapy, osteopathic manipulative therapy, reflexology, Reiki, spiritual healing, TCM, yoga, and taking dietary supplements are all examples of CAM, according to the NCCAM. Nine medical specialties, including acupuncture, TCM, homeopathy, homotoxicology, Ayurvedic medicine, anthroposophic medicine, phytotherapy, osteopathy, and chiropractic, have been defined by the National Federation of Medical Doctors and Dentists in Europe in 2002 as making up CAM therapy [3].

Acupuncture

Although acupuncture has been practiced for thousands of years in China and other parts of Asia, it was first described in print in 2,600 BC by the Yellow Emperor Hung Ti in his book of medicine. Acupuncture is defined as the insertion of a solid needle into any area of the human body to treat, prevent, or maintain health. An example of this is trigger-point acupuncture, in which needles are used to relax inflammatory muscle fibers far from the problem’s cause. Needles are inserted into the skin in a number of sites [4]. Acupuncture works by stimulating the body at particular locations. With the aid of moderate electrical stimulation, acupuncture therapy entails carefully manipulating the troublesome areas with thin steel needles. You can also warm or press these concepts (acupressure) (moxibustion). Acupuncture can reportedly treat the majority of the body’s systems, according to research [5]. In general, acupuncture works by boosting the neurological system’s ability to change how it responds to pain signals by releasing endorphins and other naturally occurring medicines. The following acupuncture effects have been identified by contemporary scientific researchers: numerous physiological processes are regulated, analgesia is produced, local microcirculation is increased, the limbic-paralimbic-neocortical network is modified, and the body is protected from infections [6]. Acupuncture is a non-specific therapy with a wide variety of indications, notably for functional problems. Its therapeutic benefits are brought on by its regulatory effects on various systems. Acupuncture has a wide range of applications in dentistry. Acupuncture can be used in prosthodontics to treat the following conditions: anxiety or nervousness, gag reflex, temporomandibular problems, postoperative dental pain, and xerostomia [7].

Postoperative dental pain: One of the more common minor surgical procedures in oral and maxillofacial surgery is the removal of impacted third molars from the jaw. It is well-recognized that this procedure may cause morbidities such as pain, edema, and trismus that are linked to a severe inflammatory reaction [8]. To manage the postoperative pain and edema associated with third molar surgery, patients are commonly given medications both before and after surgery. Non-steroidal anti-inflammatory drugs (NSAIDs) commonly suppress the cyclo-oxygenase enzyme. However, these medications have also been linked to an increased tendency to bleed, stomach ulcers, and other side effects. Kitade and Ohyabu looked at the analgesic effects of electroacupuncture (EA) on pain following third molar surgery [8]. Depending on how difficult it was to extract the tooth, they discovered either a larger tendency for pain, though not a significant increase, or a decrease in discomfort. Additionally, the researchers found that compared to postoperative EA treatments, preoperative and postoperative EA therapies had a somewhat higher analgesic effect. The effectiveness of EA in treating postoperative pain after mandibular third molar surgery was examined by researchers [9]. Depending on how difficult it was to extract the tooth, the researchers discovered either an increased tendency for pain, though not a substantial increase, or a decreased tendency for pain. Additionally, the researchers found that compared to postoperative EA treatments, preoperative and postoperative EA therapies had a somewhat higher analgesic effect. The effectiveness of EA in treating postoperative pain after mandibular third molar surgery was examined by Tavares et al. [10].

Gag reflex: One of the most striking and effective examples of the potential of acupuncture is its ability to help dental patients control their strong gag reflex. There are various methods for treating the disease, ranging from distraction to intravenous sedation. On the other hand, acupuncture offers a rapid, simple, and predictable way to control the reaction with few adverse effects. Although there are many possible locations, ear acupuncture has excelled in research. Additionally, arm points and a location called CV24 (conception vessel) in the labiomental fold on the chin. Studies have revealed that CV has an efficiency rate of more than 80% in aiding impression creation.

The needles are usually manipulated before the treatment for 30 seconds and then left in situ during treatment. Acupuncture of point pericardium 614 (PC-6) located on the forearm is considered an important point for the reduction of the gag reflex. A concave area present between the first and second metacarpal bones, commonly called Hegu cave36, is another anti-gagging point [11].

Nervousness or anxiety: Dental anxiety which is a frequent issue in dentistry can be successfully managed with acupuncture. Needling at the location on the vertex a little posterior to the vertex of the head may help treat mild-to-moderate anxiety. More hand and foot points as well as four additional needles are frequently employed. Within two minutes, relaxation can begin [12].

Temporomandibular disorders: Both acute and chronic temporomandibular disorders (TMDs) may respond effectively to acupuncture treatment. Local acupuncture points, such as those in the head and neck, are frequently employed, although, in some circumstances, distant points on the hands and feet may also be used. These result in analgesia, a decrease in muscular spasm, and can also stop the TMJ from clicking because needling the lateral pterygoid muscle can stop these muscles from spasming, which reduces the anterior displacement stresses on the joint’s meniscus. Additionally, because stress is frequently linked to TMD, the abovementioned location might be used to reduce the patient’s overall anxiety, hence easing TMD symptoms [13].

Xerostomia: Receiving prosthetic therapies may be difficult for xerostomia patients. The ability of saliva to lubricate removable dentures is crucial for comfort and retention because dry mucosa can make it difficult for the prosthesis to stay in place. Saliva flow aids in mastication, the creation of food boluses, and swallowing, in addition to being essential for speech and articulation. Auricle, digital, and local points on the face are acupuncture sites that can be used to treat xerostomia [13]. Regional acupuncture points Daying, Jiache, and Xiaguan are used on the face. During and after acupuncture treatment, patients with severe xerostomia have been shown to have significantly higher salivary flow [13,14].

Neural disorders: Acupuncture can be used to treat a wide range of neurological conditions, including facial palsy, trigeminal and other neuralgias, post-herpetic neuralgias, and lower lip paresthesia or anesthesia following lower-third molar extraction. Acupuncture is used to treat Bell’s palsy because it is believed by TCM that inserting needles at both close and far places can regulate Qi flow in the meridians, harmonize Qi-blood balance, and strengthen the body’s defense against pathogens of the External Wind [15]. Acupuncture may also encourage the regeneration of nerve fibers and neuronal excitability. According to some studies, there may be a connection between acupuncture and the autonomic nervous system. The protrusion of the masseter muscle near the angle of the jaw and the depression between the zygomatic arch and the mandibular notch are two regional acupuncture points utilized for facial palsy. These two places are physically close to the facial nerve branches. Patients with trigeminal neuralgia received daily low-frequency EA treatments for 10 days in a study. Three sessions of the treatments were given, each with a week in between. Two patients’ therapies failed, four patients only obtained partial alleviation, and 36 patients recovered fully. The following are a few benefits of acupuncture: it is secure because it is harmless, compared to many other traditional treatment procedures, there are few adverse effects, and there is no drug dependence as seen with narcotics, and it is simple and useful if done by a qualified specialist. The following are some disadvantages of acupuncture: more time commitment, often complete analgesia may not be attained, because few will be able to tolerate the needling, children should not use this, unable to be used successfully with people who are needle-phobic, there is not enough data or evidence from science [15].

Acupressure

Acupressure stimulates the points with moderate finger pressure as opposed to tiny needles used in acupuncture, making them less invasive treatments. During impression procedures, the acupressure point Chengjiangis helps in controlling the gag reflex. Between the chin and the lower lip, it is located in the mentolabial groove. Put a little pressure with your index finger. Gradually, increase the finger pressure until the patient feels uncomfortable and congested [16]. The acupressure technique must start at least five minutes before taking the impression, should be continued during the impression-taking process, and should be terminated immediately after the patient’s impression has been removed from their mouth [16].

Homeopathy

One of the most often used alternative therapies nowadays is homeopathy. According to legend, Christian Friedrich Samuel Hahnemann is credited with founding homeopathy [17]. The homeopathic medicines he developed were first printed in the Materia Medica in 1927. The word homeopathy comes from the Greek words *homios *(similar) and *pathos *(suffering or illness). The basis of homeopathy is the law of similars. According to the law, a chemical can treat a disease if it makes healthy people experience symptoms that are comparable to but much less severe than those of the illness [17]. By enhancing the immune system or vital energy, homeopathy helps the body heal itself and conquer disease or disharmony. Homeopathy is useful in prosthodontics for treating a number of ailments, including TMDs, nervousness or anxiety, denture ulceration, and xerostomia. Table 1 lists various homeopathic treatments that can be utilized in prosthodontics.

**Table 1:** Various homeopathic remedies which can be used in prosthodontics. [17].

Conditions	Homeopathic medicines
Denture ulceration	Mercurius cyanatus, Arsenium album
Nervousness or anxiety	Gelsemium, Lycopodium
Temporomandibular disorders	Ammonium carbonica, Causticum
Xerostomia	Aconite, Muriaticum

Ayurveda/Herbal Medicines

The history of Ayurveda spans from 4500 to 1600 BC. One of the greatest gifts from the sages of ancient India to humanity is Ayurveda. Natural therapeutic qualities can be found in ayurvedic herbs. The balance of the body is maintained by using the right herb in the right combination. Herbal remedies in prosthodontics are beneficial for the following ailments: denture ulceration, nervousness or anxiety, TMDs, and xerostomia. Various ayurvedic and herbal medications that can be utilized in prosthodontics are listed in Table 2.

**Table 2:** Various ayurvedic and herbal medications that can be utilized in prosthodontics. [17].

Conditions	Ayurvedic/Herbal medicines
Denture ulceration	Commiphora molmol (Myrrha), Acacia catechu (Cutch tree), Glycyrrhiza glabra (Licorice)
Nervousness or anxiety	Nepata cataria (Catnip), Saliva officinalis (Sage), Stachys officinalis (Wood betony)
Temporomandibular disorders	Articum lappa (Burdock), Symphytum officinale (Comfrey), Datura metel (Thorn apple)
Xerostomia	Pilocarpus, various species (Jaborandi), Echinacea, various species (Echinacea), Oenothera biennis (Evening primrose)

Hypnosis

Since there have been records of human activity, hypnosis has been practiced under many different names. One of the earliest types of therapy is suggestion therapy. The earliest known instance of clinical hypnosis comes from around 1773. Around 1843, James Braid, MD, coined the term hypnosis. Hypnosis is a term used to describe an altered awareness, consciousness, or perception [18]. The patient’s conscious and unconscious thoughts are both focused and receptive to therapeutic recommendations in this extremely calming condition. The following disorders can be treated primarily with hypnosis in prosthodontics: gag reflex, nervousness or anxiety, adaptation to new dentures, and control of salivation. Some patients have anxiety at the thought of having their impressions taken, both because of the potential for retching as well as the potential for personal shame. By minimizing the extra sensitivity that contributes to those uncomfortable experiences, certain hypnotic suggestions directed to the soft palate and upper throat can help with this unpleasant sensation. Additionally, hypnosis can help a patient unwind and temporarily eliminate or reduce the gag reflex, enabling dental treatment [18].

Nervousness or anxiety: It has been discovered that 8-15% of people are afraid about their dental state [18]. Psychologically, some patients perceive the oral cavity as a region where the dentist intrudes into the patient’s body, causing them to be anxious when seeking dental care. Kirsch, Montgomery, and Sapirstein conducted research in the United States and found that hypnosis compares to other psychological treatments, indicating that hypnotic interventions are becoming increasingly strong and therapeutically helpful in modern dentistry [18]. Hypnotic techniques, combined with pertinent therapeutic suggestions, assist them in relaxing and, in addition, offer them an auto-hypnosis routine to apply at home, strengthening the coping strategies provided during one-on-one sessions with the therapist.

Getting used to new dentures: One of the issues that all prosthodontists face at some point in their careers is securing adequate cooperation in the wearing of newly delivered dentures. In these circumstances, hypnosis is used to boost motivation, provide appropriate recommendations to improve tolerance, and facilitate the proper attitude toward any level of discomfort.

Salivation control: Patients who salivate excessively can pose a challenge to dentists. Hypnosis is helpful for reducing salivary flow for the amount of time required to complete the task at hand [18].

Nutritional Therapy

Traditional dentists have historically used surgery to treat gum disease, while holistic dentists view tooth decay and gum disease as external symptoms of underlying body chemistry imbalances that should be treated with nutrition treatment. Additionally, nutrition therapy is used to treat chronic infections from root canals, which holistic dentists believe are related to arthritis, heart disease, kidney and bladder issues, and mercury buildup from amalgam fillings. For the majority of dental-related conditions, dentists suggest vitamin and mineral supplements. According to a study, appropriate doses of certain vitamins and minerals, such as B6, zinc, or copper, can frequently ease pain within hours [19]. Additionally, vitamin C, copper, calcium, magnesium, and zinc can also help to lessen many gum disease symptoms. How can a dentist determine whether a patient has this imbalance? Some dentists would rather refer the patient to a nutritionist rather than participate in nutritional advice [19]. The dentist is advised to send a small sample of hair for tissue mineral analysis to a lab that will then provide a tailored supplementation and dietary plan to first become aware of their own vitamin and mineral shortages and imbalances. The dental patient’s nutritional status can then be evaluated to see if it is sufficient to fend against gum disease and tooth decay. Patients at dental offices are required to complete a questionnaire detailing their daily diet of main foods and sweets [20]. The patient can read this analysis to get clear and specific information about their illness and nutrient imbalance. Additionally, it includes a customized supplement regimen as well as a detailed eating schedule with menus. Because the majority of patients take vitamins and minerals, they frequently want to know which ones, in what doses, and which foods are ideal for their particular chemistry [20].

Medical practices that are not a part of a specific medical system are referred to as alternative therapies (AT). ATs were first observed outside of CM, and through practice or observation, their potential medicinal utility was later discovered. The following ATs have been acknowledged by and are now being used by CM and CAM. Music-assisted therapy (MuAT) was only developed in North America in the late 18th century, despite the fact that music has been utilized as a therapeutic technique since ancient times [21]. In addition to improving mood and psychological well-being, the unique ability of MuAT to alter attitude enables it to boost physiological balance, health, rehabilitation, and sickness prevention. As a result, society and the medical profession are gradually embracing it. A certified music therapist performs MuAT to accomplish specified goals using music interventions and music therapy practices. Music therapists employ music in all of its physical, emotional, cerebral, social, aesthetic, and spiritual components to help clients achieve or maintain health. Music interventions include making musical instruments, playing instruments like the piano and drums, singing songs, analyzing lyrics, creating music, and creating lyrics. To assist well-being and/or stress management, pain alleviation, emotion expression, memory improvement, communication improvement, and physical rehabilitation, interventions can be developed. Additionally, music therapy can be used to focus on children’s and young adults’ developmental stages or to distract patients from uncomfortable symptoms by integrating them into educational settings. The importance of music in developing a complete approach that focuses on a child's or young person’s developmental stage is highlighted in this passage. Health use research has indicated that MuAT is helpful in a variety of therapeutic scenarios, despite the fact that it is still in the early phases of development [21].

The neonatal intensive care unit (NICU) now provides music therapy as a way to improve treatment and encourage the growth and development of preterm newborns [22]. Aggression in children is becoming a major concern due to the rise in youngsters reporting aggressive and violent conduct. Although the antipsychotic drug risperidone, a common drug, is effective in treating children’s aggressive behavior, it also has some negative side effects, including increased appetite, lethargy, drowsiness, dizziness, and drooling. Music has been shown to reduce violent behavior and increase self-esteem in children who have strong aggressive tendencies. Music therapy is recommended for people of all ages who suffer from a variety of conditions, including psychiatric disorders, medical conditions, physical impairments, sensory impairments, developmental disabilities, substance abuse, communication disorders, interpersonal issues, and dementia-related aging. Music therapy may help with the treatment of physiological stress, physical stress, and combined physiological/physical stress response, as well as postoperative pain, particularly the pain felt by breast cancer patients after a radical mastectomy. Cardiovascular rehabilitation and bone marrow/stem cell transplantation are some of these additional illnesses or ailments. Currently, MuAT is provided in hospice care, and music therapists routinely participate in care teams in hospitals and nursing homes. When a patient is reaching the end of their life, music therapy is utilized to improve their quality of life by helping to relieve their symptoms, tend to their psychological needs, offer support, encourage communication, and tend to their spiritual needs. Additionally, it is used to promote a range of other health-related activities, minimize loneliness and depressive symptoms, reduce stress, increase physical exercise, and help terminally ill patients convey their thoughts and feelings [23]. Studies have revealed that music has positive effects on animals in addition to having significant positive effects on people. For instance, studies on the effects of music therapy on immune function parameters and hypothalamic-pituitary-adrenal axis responses revealed that it may have therapeutic benefits for asthmatic rats that experienced stress early on. Music was played continuously for 21 days (six hours per day at low sound pressure levels of 50-60 dB) on 131 mice [24]. This resulted in higher levels of brain-derived neurotrophic factor and lower levels of nerve growth factor in the hypothalamus. Listening to music reduced levels of ovarian hormones (mostly progesterone) and anxiety in female mice. Music therapy significantly reduced anxiety in BDNF (Met/Met) mice, and blood interleukin-4 and corticosterone in ovalbumin-induced asthmatic rats. When dairy cows are being milked, soothing music enhances milk output. Medical schools can incorporate CM training into their curricula, lowering the cost of getting the therapeutic effects of music. Interdisciplinary hospice staff can be trained in the use of a MuAT protocol through MuAT courses. The most recent studies demonstrating the positive benefits of MuAT suggest that it might be an advantageous addition to both conventional and integrative medical treatments. MuAT must be customized for each patient because different musical genres might have various physiological and psychological effects on people. It can be assumed that music has a bright future in human healthcare because MuAT offers a holistic approach to patients with significant physiological and psychological benefits or those with particular arousal due to stress and because it is an affordable, nonpharmacological, and noninvasive therapy.

The use of an animal to improve human health or well-being is known as animal therapy or animal-assisted interventions (AAI). AAI now includes service-animal programs, animal-assisted activities, and animal-assisted therapy (AAT) [25]. AAT for patients with mental disorders was first documented in the late 18th century. Animals with certain traits that make them fundamental are used in therapies or activities. For various forms of therapy, a variety of animals, such as dogs, cats, birds, rabbits, birds, lizards, or other tiny creatures, may be employed. AAT has also occasionally utilized horses, virtual pets (for patients with autism), dolphins (for patients with CP), and elephants (for patients with disabilities). There are situations when toy animals can provide advantages to humans. AAT or AAA provides motivational, educational, and/or recreational benefits in promoting quality of life by improving the physical, social, emotional, and/or cognitive functioning of patients and/or participants. AAT is typically delivered by trained professionals or volunteers who are familiar with the patients involved as well as with animals in general. Depending on the patient’s needs and the therapy’s objectives, it could entail either brief or extended patient-animal interactions. In some circumstances, the patient can end up taking care of the animal. Considerable research has shown how interactions with companion animals improve human health. Therapy and behavioral interventions are required for many disabled children, but CM is not always successful for them. Animal therapy might be advantageous for them. In Europe, horseback riding treatment is a well-liked physical rehabilitation, whether it involves a real horse or a robotic horse (a home machine). In a 2009 study, compared to autistic children without therapeutic horseback riding (n = 15), nine autistic children showed greater sensory seeking, sensory sensitivity, and social motivation [26-28]. They also showed less inattention, distractibility, and sedentary habits. When compared to time spent on a waiting list, other studies have indicated that this type of therapy helps children with CP with their gross motor function. In children with enuresis and other psychoneurological symptoms, dolphin therapy has been demonstrated to have a good clinical impact. In a study with 5,741 participants, it was discovered that pet owners had considerably lower lipid and blood pressure levels than non-pet owners [28].

A relatively recent area of medicine is AATs, which aim to improve human well-being. In this area, there is still much to be done, especially with regard to the protection of animal welfare and the morality of using animals in healthcare. Despite the rise in pet ownership in the United States, little attention has been paid to AAT in terms of pertinent research, education, and practice. Robots that resemble humans have been created thanks to recent technological advancements, and they are being used in households as well as factories. According to research, for some psychological or physical disorders, robot-assisted therapy may be just as successful as AAT. Therefore, therapies involving virtual pets or those supported by robots can address ethical and animal welfare concerns [29-34]. AAT can be a helpful therapy option for some illnesses that can be incorporated into a patient’s plan of care as a recognized therapeutic modality given the advantages proven by current AAT research [35].

## Conclusions

Alternative therapies should be viewed as a complement to traditional treatment in general. Its effectiveness as a stand-alone therapy is debatable, but when paired with conventional treatment, it could be a valuable addition to the prosthodontist therapeutic arsenal. Alternative therapy is here to stay, and it is no longer an option to dismiss it or treat it as something outside of science and medicine’s usual processes. The task at hand is to proceed with caution, utilizing both reason and wisdom to separate the pearls from the dirt. Traditional medicine has a tried-and-true method called alternative therapies. Alternative treatments have been utilized for thousands of years in China and other Eastern countries to encourage and maintain healthy dental health. Because alternative therapies are typically safe, nontoxic, and produce very few side effects, they can be used in conjunction with traditional therapy approaches to reduce patient anxiety in dental offices.

## References

[1] Amruthesh S (2008). Dentistry and Ayurveda - IV: classification and management of common oral diseases. Indian J Dent Res.

[2] Kumar S, Yadav R, Yadav V, Sidhu MS, Prabhakar M, Dabas A (2017). Alternative therapies and prosthodontics: a combined approach. Indian J Health Sci Care.

[3] Blom M, Dawidson I, Angmar-Månsson B (1992). The effect of acupuncture on salivary flow rates in patients with xerostomia. Oral Surg Oral Med Oral Pathol.

[4] Naik PN, Kiran RA, Yalamanchal S, Kumar VA, Goli S, Vashist N (2014). Acupuncture: an alternative therapy in dentistry and its possible applications. Med Acupunct.

[5] Jiang Y, Wang H, Liu Z (2013). Manipulation of and sustained effects on the human brain induced by different modalities of acupuncture: an fMRI study. PLoS One.

[6] Ernst E, Pittler MH (1998). The effectiveness of acupuncture in treating acute dental pain: a systematic review. Br Dent J.

[7] Stux G, Pomeranz B (1995). Basics of Acupuncture. Oral Dis.

[8] Lao L, Bergman S, Langenberg P, Wong RH, Berman B (1995). Efficacy of Chinese acupuncture on postoperative oral surgery pain. Oral Surg Oral Med Oral Pathol Oral Radiol Endod.

[9] (1998). NIH Consensus Conference. Acupuncture. JAMA.

[10] Rosted P (2000). Introduction to acupuncture in dentistry. Br Dent J.

[11] Vachiramon A, Wang WC (2002). Acupressure technique to control gag reflex during maxillary impression procedures. J Prosthet Dent.

[12] Arabzade Moghadam S, Yousefi F, Saad S (2021). The effect of hypnosis on pain relief due to injection of dental infiltration anesthesia. Clin Exp Dent Res.

[13] Johnstone PA, Niemtzow RC, Riffenburgh RH (2002). Acupuncture for xerostomia: clinical update. Cancer.

[14] Blom M, Lundeberg T (2000). Long-term follow-up of patients treated with acupuncture for xerostomia and the influence of additional treatment. Oral Dis.

[15] Linde K, Alscher A, Friedrichs C, Joos S, Schneider A (2014). [The use of complementary and alternative therapies in Germany - a systematic review of nationwide surveys]. Forsch Komplementmed.

[16] Ulett GA, Han S, Han JS (1998). Electroacupuncture: mechanisms and clinical application. Biol Psychiatry.

[17] Michalek-Sauberer A, Heinzl H, Sator-Katzenschlager SM, Monov G, Knolle E, Kress HG (2007). Perioperative auricular electroacupuncture has no effect on pain and analgesic consumption after third molar tooth extraction. Anesth Analg.

[18] Kirsch I, Montgomery G, Sapirstein G (1995). Hypnosis as an adjunct to cognitive-behavioral psychotherapy: a meta-analysis. J Consult Clin Psychol.

[19] Purohit A, Singh A, Purohit B, Shakti P, Shah N (2021). Is aromatherapy associated with patient's dental anxiety levels? A systematic review and meta-analysis. J Dent Anesth Pain Med.

[20] Gao X, Hamzah SH, Yiu CK, McGrath C, King NM (2013). Dental fear and anxiety in children and adolescents: qualitative study using YouTube. J Med Internet Res.

[21] Zabirunnisa M, Gadagi JS, Gadde P, Myla N, Koneru J, Thatimatla C (2014). Dental patient anxiety: possible deal with lavender fragrance. J Res Pharm Pract.

[22] Worwood VA (2016). The Complete Book of Essential Oils and Aromatherapy, Revised and Expanded: Over 800 Natural, Nontoxic, and Fragrant Recipes to Create Health, Beauty, and Safe Home and Work Environments. New World Library.

[23] Fischer-Rizzi S (1990). Complete Aromatherapy Handbook: Essential Oils for Radiant Health. https://books.google.co.in/books?hl=en&lr=&id=43HyCe20IvMC&oi=fnd&pg=PA7&dq=Complete+aromatherapy+handbook:+Essential+oils+for+radiant+health&ots=Oc6-KRDBuf&sig=HFuTCUMNTm45dBsDopffjTU1Y-w&redir_esc=y#v=onepage&q=Complete%20aromatherapy%20handbook%3A%20Essential%20oils%20for%20radiant%20health&f=false.

[24] Astin JA, Marie A, Pelletier KR, Hansen E, Haskell WL (1998). A review of the incorporation of complementary and alternative medicine by mainstream physicians. Arch Intern Med.

[25] Jimson S, Malathi L, Devi N, Sankari S (2016). Aromatherapy in dentistry - a review. Biomed Pharmacol J.

[26] Chouhan S, Sharma K, Guleria S (2017). Antimicrobial activity of some essential oils-present status and future perspectives. Medicines (Basel).

[27] Lim MA, Borromeo GL (2017). The use of general anesthesia to facilitate dental treatment in adult patients with special needs. J Dent Anesth Pain Med.

[28] Lehrner J, Marwinski G, Lehr S, Johren P, Deecke L (2005). Ambient odors of orange and lavender reduce anxiety and improve mood in a dental office. Physiol Behav.

[29] Rashidi Maybodi F, Jalali Pandary M, Karami E, Ebrahimi AR (2018). The effect of music and lavender's aroma on patients anxiety, during periodontal surgery. J Dent Mater Tech.

[30] Venkataramana M, Pratap KV, Padma M, Kalyan S, Reddy AA, Sandhya P (2016). Effect of aromatherapy on dental patient anxiety: a randomized controlled trial. Indian Assoc Public Health Dent.

[31] Soni S, Bhatia R, Oberoi J (2018). Evaluation of the efficacy of aromatherapy on anxiety level among pediatric patients in a dental setting: a randomized control trial. Int J Oral Care Res.

[32] Pradopo S, Sinaredi BR, Januarisca BV (2017). Pandan leaves (Pandanus amaryllifolius) aromatherapy and relaxation music to reduce dental anxiety of pediatric patients. J Int Dent Med Res.

[33] Nardarajah D, Dhanraj M, Jain AR (2018). Effects of lavender aromatherapy on anxiety levels of patients undergoing mandibular third molar extraction. Drug Invent Today.

[34] Hasheminia D, Kalantar Motamedi MR, Karimi Ahmadabadi F, Hashemzehi H, Haghighat A (2014). Can ambient orange fragrance reduce patient anxiety during surgical removal of impacted mandibular third molars?. J Oral Maxillofac Surg.

[35] Kritsidima M, Newton T, Asimakopoulou K (2010). The effects of lavender scent on dental patient anxiety levels: a cluster randomised-controlled trial. Community Dent Oral Epidemiol.

